# Adenovirus-mediated artificial miRNA targetting fibrinogen-like protein 2 attenuates the severity of acute pancreatitis in mice

**DOI:** 10.1042/BSR20170964

**Published:** 2017-11-23

**Authors:** Xiaohua Ye, Jin Ding, Yanping Chen, Jiayue Dong

**Affiliations:** Department of Gastroenterology, Jinhua Hospital of Zhejiang University, Jinhua 321000, China

**Keywords:** acute pancreatitis, apoptosis, fibrinogen-like protein 2, molecular mechanism

## Abstract

Severe acute pancreatitis (SAP) remains to be challenging for its unpredictable inflammatory progression from acute pancreatitis to SAP. Apoptosis is an important pathology of SAP. Fibrinogen-like protein 2 (FGL2) has been reported to be involved in apoptosis. The present study aimed to explore the therapeutic effect of an adenovirus-mediated artificial miRNA targetting FGL2 (Ad-FGL2-miRNA) in taurocholate-induced murine pancreatitis models. Sodium taurocholate was retrogradely injected into the biliopancreatic ducts of the C57/BL mice to induce SAP. FGL2 expression was measured with reverse transcription-PCR, Western blotting, and immunohistochemical staining. ELISA was used to detect the activity of amylase and the concentrations of tumor necrosis factor-α (TNF-α) and interleukin-1β (IL-1β). In addition, the mRNA levels of TNF-α and IL-1β were also detected. Finally, apoptosis was assessed by terminal deoxynucleotidyl transferase mediated dUTP-biotin nick-end labeling (TUNEL) method and Western blotting. Ad-FGL2-miRNA significantly suppressed FGL2 expression and alleviated pancreatic injury. Also, Ad-FGL2-miRNA markedly inhibited a post-SAP increase in the activation of TNF-α and IL-1β. Finally, pretreatment with Ad-FGL2-miRNA ameliorated apoptosis at the early stage of SAP by modulating cleaved caspase-3 and therefore played a protective role. These results indicated that FGL2 might be a promising target for attenuating the severity of SAP and adenovirus-mediated artificial miRNAs targetting FGL2 represented a potential therapeutic approach for the treatment of SAP.

## Introduction

Severe acute pancreatitis (SAP) is a potentially fatal pathogenic condition characterized by rapid progression and high mortality [1], but the underlying pathophysiological mechanisms remain incompletely defined. The death of pancreatic acinar cell is the major pathophysiological change in early onset of acute pancreatitis, and the modalities of cell death generally contain necrosis and apoptosis [1–3]. The damage of pancreatic acinar cells during SAP is usually through the interaction of apoptosis and necrosis [2]. Recent studies have revealed that mild acute pancreatitis (MAP) is mainly associated with apoptotic acinar cell death, whereas necrosis plays a predominant role in SAP with a severe inflammatory response [2]. Although cell death is unlikely to be favorable, a transformation from necrosis to apoptosis in response to a pathologic stimulus can attenuate experimental pancreatitis less severe and thus confers a beneficial effort [3].

In the recent years, accumulating studies have provided strong evidence in supporting a pivotal role of toll-like receptor 4 (TLR4) and p38 mitogen-activated protein  kinase (MAPK) signaling in the pathogenesis of SAP [4–6]. TLR4 is a kind of inflammatory transmembrane receptor that is expressed by epithelia in pancreatic ducts and acinus. TLR4 activated downstream signal transduction pathway during the development of SAP, thereby regulating inflammation, cell survival, and apoptosis [5]. p38 MAPK, one of the three major families of MAPKs (p38 MAPK, Erk 1/2, and JNK), is up-regulated during SAP by induction of proinflammatory  cytokines  and  stress  stimuli [6].

Fibrinogen-like protein 2 (FGL2) was identified as a new member of the fibrinogen-related protein superfamily (fibrinogen-related domain), which includes fibrinogen, tenascin, ficolin, and angiopoietin [7]. FGL2 has two structurally different forms with distinct properties: the membrane-bound FGL2 and the soluble FGL2. Membrane-bound FGL2 is a novel procoagulant that mainly leads to histopathological lesions and ischemic injury by mediating ‘immune coagulation’, fibrin deposition, and microthrombosis [8,9]. Soluble FGL2 is the other form that is highly secreted by regulatory T cells (Treg) and presents contradictory properties in tissue injuries [10–12]. Previous studies show that soluble FGL2 is involved in Treg activity and demonstrates immunoregulatory function to prevent tissue damages [7,11]. Moreover, FGL2 has also been reported to facilitate cellular apoptosis, including sinusoidal endothelial cells and hepatocytes through binding to its inhibitory FcγRIIB receptor on the cell surface and results in tissue injuries [13,14]. In addition, targetted deletion of FGL2 ameliorated hepatocyte necrosis and apoptosis in MHV-3-induced fulminant hepatitis in mice [15]. Our previous studies found that the expression of FGL2 was elevated in both SAP animal model and patients [16–18]. These results are preliminary, but very meaningful. As we know, SAP is closely related to apoptosis. Therefore, it allows us to hypothesize whether FGL2 plays a pivotal role in the apoptotic mechanism of SAP.

In the present study, we constructed a recombinant adenovirus that encoded an artificial miRNA specifically targetting FGL2 (Ad-FGL2-miRNA) and aimed to evaluate its therapeutic effect in SAP mice and its possible molecular mechanism involved.

## Materials and methods

### Animals

Seventy-two specific pathogen free male C57/BL mice were purchased from Research Science Biotechnology Co., Ltd (Shanghai, China). All animals were fed standard chow, had free access to water, and were housed in a room with constant temperature of 25°C and a 12-h day/night cycle. All animals were acclimated for at least 1 week before the experiments were initiated. All procedures were performed in accordance with the Guidelines of Animal Care and Use Committee of Jinhua Hospital of Zhejiang University.

### Transfection with Ad-FGL2-miRNA

Recombinant adenovirus carrying miRNA against FGL2 was designed and produced by Research Science Biotechnology Co., Ltd (Shanghai, China). The double-stranded miRNA templates for FGL2 were inserted into the pAdeno-U6-CMV-EGFP vector, and recombinant siRNA plasmids were transfected into 293T cells (ATCC, Manassas, VA, U.S.A.). The oligonucleotides encoding FGL2-miRNA sequences were listed as below: top: 5′-TGCTGTTCTTTGAGCACCTCCTCCATGTTTTGGCCACTGACTGACATGGAGGATGCTCAAAGAA-3′, bottom: 5′-CCTGTTCTTTGAGCATCCTCCATGTCAGTCAGTGGCCAAAACATGGAGGAGGTGCTCAAAGAAC-3′. C57/BL mice were injected intravenously in the tail with 0.5 ml of 4 × 10^8^ PFU/ml Ad-FGL2-miRNA. At different prespecified time points (1, 4, and 8 h), the mice were killed by exsanguination.

### Induction of SAP

All mice received intraperitoneal injection of 10% chloraldurate (2 ml/kg body weight; Solarbio, Beijing, China) for anesthesia. The mice were divided into four groups. In the first group (sham operation (SO)), the mice underwent surgery but were not infused with 4% sodium taurocholate. In the second group (SAP), a laparotomy was performed through a midline incision. Sodium taurocholate (4%; 1 ml/kg body weight; Sigma, St. Louis, MO, U.S.A.) was retrogradely injected into the biliopancreatic duct through the papilla using a segmental epidural catheter via a microinjection pump at a speed of 0.2 ml/min. A microclip was placed in the hepatic portion of the biliopancreatic duct to avoid reflux before injection. In the third group (SAP + Ad-negative-miRNA), the mice were injected with 0.5 ml of 4 × 10^8^ PFU/ml of negative-miRNA through the tail vein 24 h before being induced in the same manner as the SAP. The fourth group of mice (SAP + Ad-FGL2-miRNA) was intravenously injected in the tail with 0.5 ml of 4 × 10^8^ PFU/ml of Ad-FGL2-miRNA 24 h prior to the injection of sodium taurocholate. After each operation, the abdomen was closed in two layers. All procedures were carried out using sterile techniques.

### Sample collection and tissue preparation

At defined time points (1, 4, 8 h; *n* = 6 per time point) after operation, mice were anesthetized with 10% chloraldurate (2 ml/kg body weight) and killed by exsanguination. Pancreatic tissues were harvested immediately and divided into two pieces. Portions of the tissues were fixed in 4% paraformaldehyde for immunohistochemical staining and microscopic observation, and other portions were removed and stored in liquid nitrogen until use. Blood samples (5 ml) were obtained via postcava puncture.

### Serum amylase activity

The blood samples were centrifuged at 1200×***g*** for 20 min, and the serum was collected for detection of amylase activity (U/dl) with an AMS test kit (Jiancheng Bioengineering, Nanjing, China).

### ELISA for tumor necrosis factor-α and interleukin-1β

Levels of serum tumor necrosis factor-α (TNF-α) and interleukin-1β (IL-1β) were determined with mouse ELISA Kit for TNF-α and IL-1β (4A Biotech, Beijing, China). Briefly, standards and samples were bound by the immobilized antibody and an enzyme-linked polyclonal antibody for TNF-α or IL-1β was added to the wells followed by a substrate solution yielding a colored product. The intensity of the color was detected at 450 nm. The sample levels were obtained from a standard curve and were corrected for protein concentration.

### Hematoxylin and Eosin staining and assessment of pancreatic tissue injury

Pancreatic tissue samples were fixed in 4% paraformaldehyde for Hematoxylin and Eosin staining. The severity of pancreatic tissue injury was evaluated in accordance with the modified method of Schmidt et al. [19]. Parameters of edema, hemorrhage, acinar cell degeneration, and interstitial inflammation were scored in ten random fields in each slide to assess the severity of pancreatic injury. Each variable was scored as follows: (i) edema: 0 = absent, 1 = focally in the interlobular space, 2 = increased in the intralobular space, 3 = isolated-island appearance of pancreatic acinus; (ii) hemorrhage: 0 = absent, 1 = slight, 2 = moderate, 3 = severe; (iii) acinar cell degeneration: 0 = absent, 1 = focal (<5%); 2 = and/or sublobular (<20%), 3 = and/or lobular (>20%); and (iv) inflammation: 0 = absent, 1 = slight, 2 = moderate, 3 = severe. The sum of these four variables for each section could reach a maximum score of 12.

### Quantitative real-time RT-PCR

Total RNA was extracted using TRIzol reagent (Takara, Dalian, Liaoning, China) according to the manufacturer’s protocols and then reversely transcribed into cDNA (MBI Fermentas, Burlington, Canada). Real-time qRT-PCR was performed on cDNA using SYBR Green (Vazyme, Nanjing, Jiangsu, China) *via* an ABI 7500 Sequence Detection System (Applied Biosystems Inc., Carlsbad, CA, U.S.A.). The sequences of the primers (Research Science) were as follows: FGL2 (194 bp): 5′-TGCCTTGCGTTTCAGTCG-3′ (forward) and 5′-AATCCCATTACGGACACCTTT-3′ (reverse); β-actin (201 bp): 5′-CCTGGAGAAACCTGCCAAGTA-3′ (forward) and 5′-TCATACCAGGAAATGAGCTTGAC-3′ (reverse); TNF-α (188 bp): 5′-GACCCTCACACTCAGATCAT-3′ (forward) and 5′-GGTACAACCCATCGGCTGGCA-3′ (reverse); β-actin (540 bp): 5′- AGGGCCCAGCACCTGCACAG-3′ (forward) and 5′-CTAGAAGCATTTGCGGTG-3′ (reverse); IL-1β (475 bp): 5′-GCTTCAGGCAGGCAGTAT-3′ (forward) and 5′-ACAAACCGCTTTTCCATCT-3′ (reverse); β-actin (218 bp): 5′- CTGTCCCTGTATGCCTCT-3′ (forward) and 5′-ATGTCACGCACGATTTCC-3′ (reverse). The cDNA was denatured at 95°C for 2 min and amplified for 40 cycles at 95°C (15 s), 60°C (20 s), and 72°C (20 s), followed by a final extension at 72°C (30 s). The samples were amplified in triplicate, and the results were calculated using the 2^−ΔΔ*C*^_T_ method. The mRNA levels of target genes were shown relative to β-actin.

### Western blotting

Pancreatic proteins were separated with SDS/PAGE (10% gel) and transferred on to a nitrocellulose membrane (Millipore, Billerica, MA, U.S.A.). The membrane was blocked with 5% skim milk in 1% TBS and then incubated with a mouse anti-FGL2 antibody (1:200 dilution; Santa Cruz Biotechnology, CA, U.S.A.), cleaved caspase-3 (1:1000 dilution; Abcam, HK, China), Bax (1:1000 dilution; EPITMICS, Burlingame, CA, U.S.A.), Bcl-2 (1:1000 dilution; Proteintech, Wuhan, China), p38 MAPK (1:1000 dilution; Cell Signaling Technology, Beverly, MA), or TLR4 (1:500 dilution; Abcam, Cambridge, MA). The membrane was washed three times with TBS and incubated with secondary antibody (Jackson ImmunoResearch, West Grove, PA, U.S.A.) conjugated to horseradish peroxidase (HRP) for 2 h at room temperature. The immunoreactive bands were visualized with an ECL reagent (Pierce, Rockford, IL, U.S.A.). Protein expression levels were normalized to β-actin.

### Immunohistochemical staining

Immunohistochemical staining was performed to assess FGL2 expression in the pancreatic tissues using EnVision reagents (Dako, Glostrup, Denmark) according to the manufacturer’s instructions. The sections were incubated with a mouse anti-FGL2 antibody (Santa Cruz Biotechnology, CA, U.S.A.; 1:50) for 2 h at 37°C. Following the same washing procedure, EnVision reagents were applied and incubated for 30 min at 37°C. Followed by incubation with HRP-labeled rabbit IgG fraction to mouse IgG Fc, the target protein was detected using a diaminobenzidine (DAB) kit (Jackson ImmunoResearch, West Grove, PA, U.S.A.). The slides were then counterstained with Hematoxylin and visualized using a microscope (Olympus, Tokyo, Japan). To measure FGL2 protein expression, ten randomly selected fields across each section were evaluated at 200× magnification.

### TUNEL

Apoptosis was assessed on pancreatic tissue by immunofluorescent terminal deoxynucleotidyl transferase mediated dUTP-biotin nick-end labeling (TUNEL, Roche, Shanghai, China) assay. Slides were treated with Proteinase K for 30 min at 37°C before being rinsed with PBS three times. Sections were left to slightly dry after the addition of permeabilization washing buffer and then incubated at room temperature for 20 min. They were then treated with the TUNEL TdT and fluorescein-labeled dUTP solution. The slides were subsequently left in the dark in a humid environment at 37°C for 60 min before washing with PBS. DAPI was then added to the slides to stain nuclei. TUNEL-stained apoptotic cells were calculated by fluorescence microscopy.

### Statistical analysis

All data represent the mean ± S.D. SPSS 15.0 software (SPSS, Chicago, IL, U.S.A.) was used for statistical analysis. The statistical significance was assessed through a one-way ANOVA. A *P*-value less than 0.05 was considered to indicate statistical significance.

## Results

### Ad-FGL2-miRNA reduced serum amylase and alleviated the histopathological alterations of the pancreas in SAP in mice

**Figure 1:**
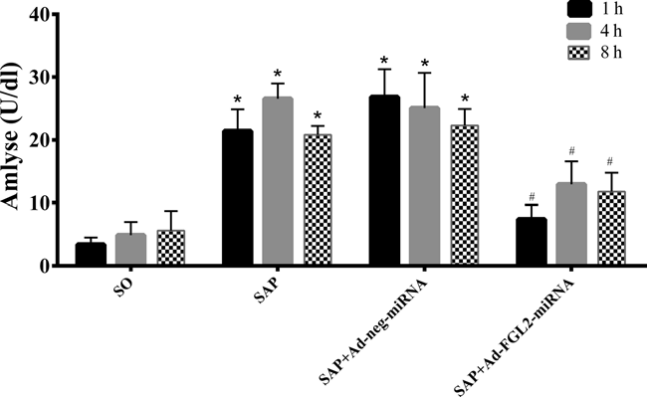
Effect of Ad-FGL2-miRNA on serum amylase in SAP Each time point (h) post operation consisted of six mice. There was no difference amongst the three time points in the SO group (*P*>0.05). The data are expressed as the mean ± S.D. *P<0.01 compared with SO group at respective time point; ^#^*P*<0.01 compared with SAP or SAP + Ad-negative-miRNA group at each time point.

Serum amylase is most commonly used as a biochemical indicator of acute pancreatitis. As shown in Figure 1, the levels of serum amylase were markedly elevated (*P*<0.01) in the SAP group compared with the SO group at each time point, and Ad-FGL2-miRNA significantly reduced the level of amylase (*P*<0.01).

**Figure 2:**
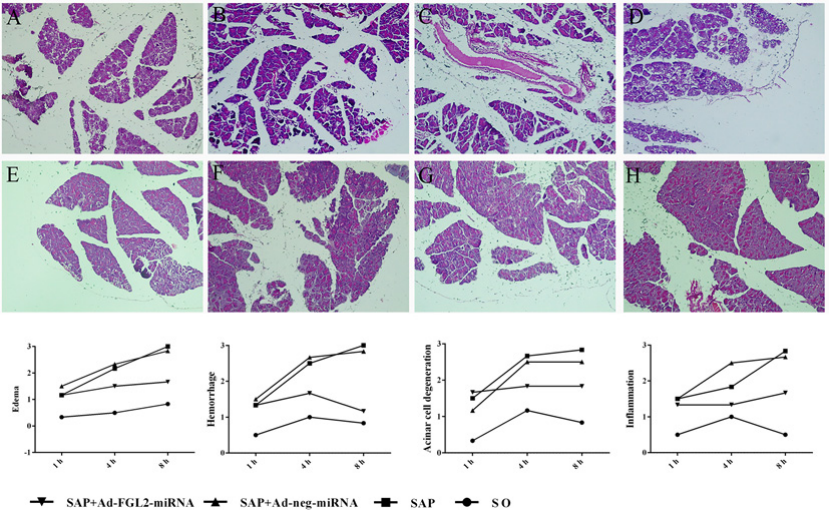
Histological changes and pathological scores for pancreatic tissues from mice in each group Each time point (h) post operation consisted of six mice. Histological changes were much more severe in the SAP and SAP + Ad-negative-miRNA groups (**A**–**D**) than in the SO (**H**) groups, but were less pronounced in the SAP + Ad-FGL2-miRNA group (**E**–**G**) at 1 h (A, E), 4 h (B, F), 8 h (C, G) after SAP induction (Hematoxylin and Eosin staining, ×200). The data are expressed as the mean ± S.D. *P*<0.01 when SAP + Ad-FGL2-miRNA group was compared with SAP and SAP + Ad-negative-miRNA groups at each time point.

We also investigated the histopathological alterations of the pancreas after administration of Ad-FGL2-miRNA. The histological features of the pancreas remained morphologically normal in SO group, whereas the pancreatic tissues in the SAP group appeared much more severely damaged. Microscopic examination of the pancreas in the SAP group showed edema, hemorrhage, acinar cell degeneration, and inflammation. Pretreatment with Ad-FGL2-miRNA markedly reduced the histological features of pancreatic injury at 1 and 4 h in SAP mice post operation (*P*<0.01). However, the severity of pancreatic damage in SAP + Ad-neg-miRNA group did not significantly alleviate. The mean pathology score for individual parameter in mice with SAP was higher (*P*<0.01) compared with normal mice at each time point (Figure 2).

### Ad-FGL2-miRNA suppressed FGL2 expression

**Figure 3:**
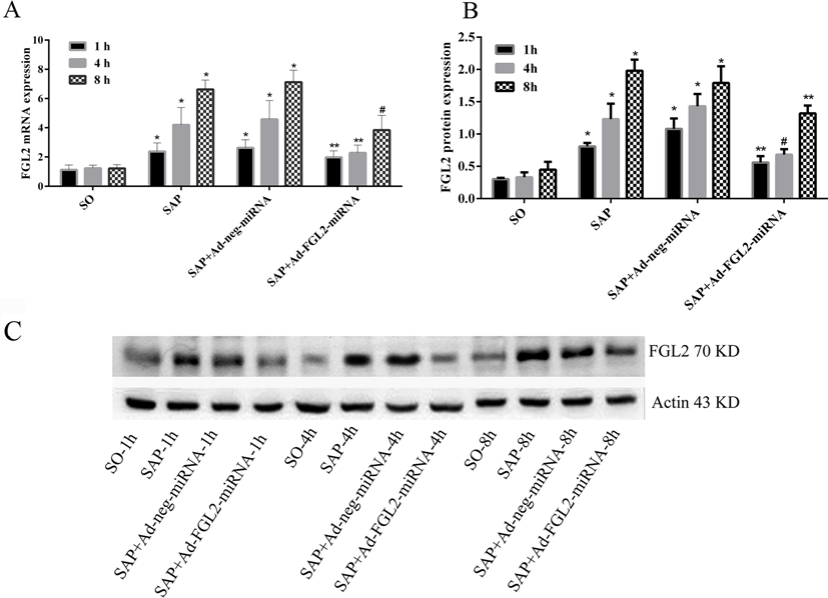
Inhibitory effect of Ad-FGL2-miRNA on the expression of FGL2 Each time point (h) post operation consisted of six mice. (**A**) Relative expression of *FGL2* mRNA in pancreas. (**B**) Relative expression of FGL2 protein in pancreas. (**C**) FGL2 expression detected by Western blotting. The data are expressed as the mean ± S.D. *P<0.01 compared with SO group at respective time point; ^#^*P*<0.01 compared with SAP or SAP + Ad-negative-miRNA group at each time point; **P<0.05 compared with SAP or SAP + Ad-negative-miRNA group at each time point.

**Figure 4:**
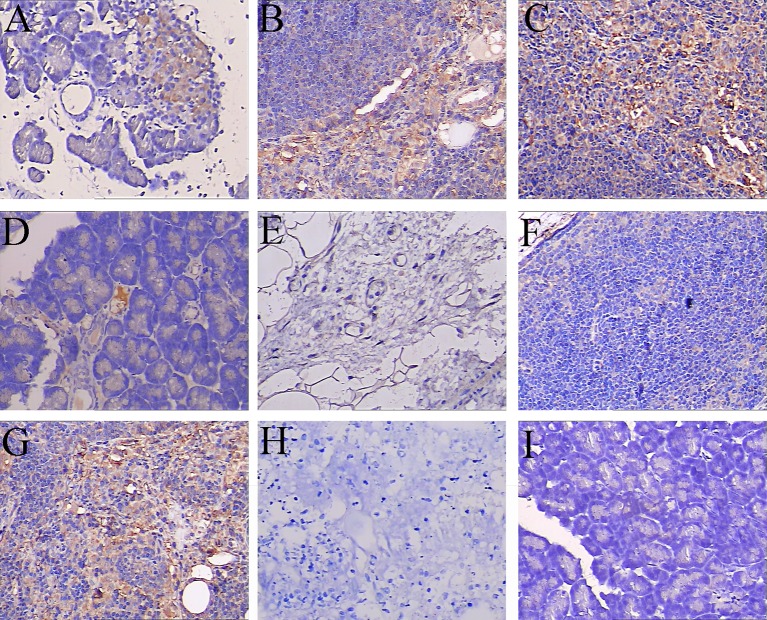
FGL2 expression detected by immunohistochemistry staining in pancreas Each time point (h) post operation consisted of six mice. FGL2 expression was significantly elevated in the SAP and SAP + Ad-negative-miRNA groups (**A**–**C, G**) than in the SO (**I**) group, but was less pronounced in the SAP + Ad-FGL2-miRNA group (**D**–**F**) at 1 h (A, D), 4 h (B, E), 8 h (C, F) after SAP induction (×200), (**H**) negative staining control.

To evaluate the inhibitory effects of Ad-FGL2-miRNA *in vivo*, a time course study was performed with real-time PCR, Western blotting, and immunohistochemical staining at defined time points. Real-time PCR revealed that the inhibitory effect of Ad-FGL2-miRNA began as soon as 1 h after SAP induction, with a maximal inhibitory effect at 4 h post establishment of the SAP model. However, the effect turned out to be less obvious at 8 h post SAP induction (Figure 3A). These results were further confirmed by Western blot analysis. The protein expression of FGL2 decreased significantly in Ad-FGL2-miRNA-treated mice (Figure 3B,C). Immunohistochemical staining demonstrated that FGL2 was primarily localized in infiltrating interstitial and endothelial cells of the microvasculature (Figure 4). Also, an increasing and aggravating tendency was found with FGL2 expression. Only low levels of FGL2 expression were detected in SO mice (Figure 4).

### Ad-FGL2-miRNA down-regulated inflammatory cytokines

**Figure 5:**
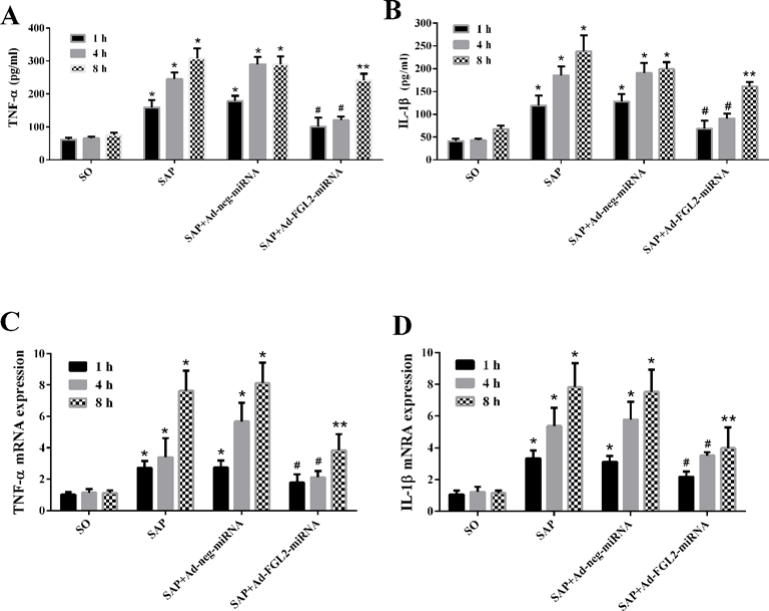
Effect of Ad-FGL2-miRNA on TNF-α (A, C) and IL-1β (B, D) in SAP. Each time point (h) post operation consisted of six mice. There was no difference amongst the three time points in the SO group (*P*>0.05). The data are expressed as the mean ± S.D. *P<0.01 compared with SO group at respective time point; ^#^*P*<0.01 compared with SAP or SAP + Ad-negative-miRNA group at each time point; **P<0.05 compared with SAP or SAP + Ad-negative-miRNA group at each time point.

To further explore the mechanisms that underlie reduced pancreatic injury after Ad-FGL2-miRNA administration, we evaluated the expression of the cytokines TNF-α and IL-1β by real-time PCR and ELISA. These cytokines play important roles in the development of SAP [4]. As shown in Figure 5, concentrations of these cytokines were similar in SO group. As indicated by both real-time PCR and ELISA, levels of IL-1β and TNF-α in SAP group began to increase at 1 h, and reached its peak at 8 h. After treatment with Ad-FGL2-miRNA, there was a significant inhibitory effect on the levels of IL-1β and TNF-α expression when compared with the SAP group (*P*<0.01). However, Ad-neg-miRNA did not show significant therapeutic effect on SAP mice.

### Ad-FGL2-miRNA prevented pancreatic apoptosis in mice

**Figure 6:**
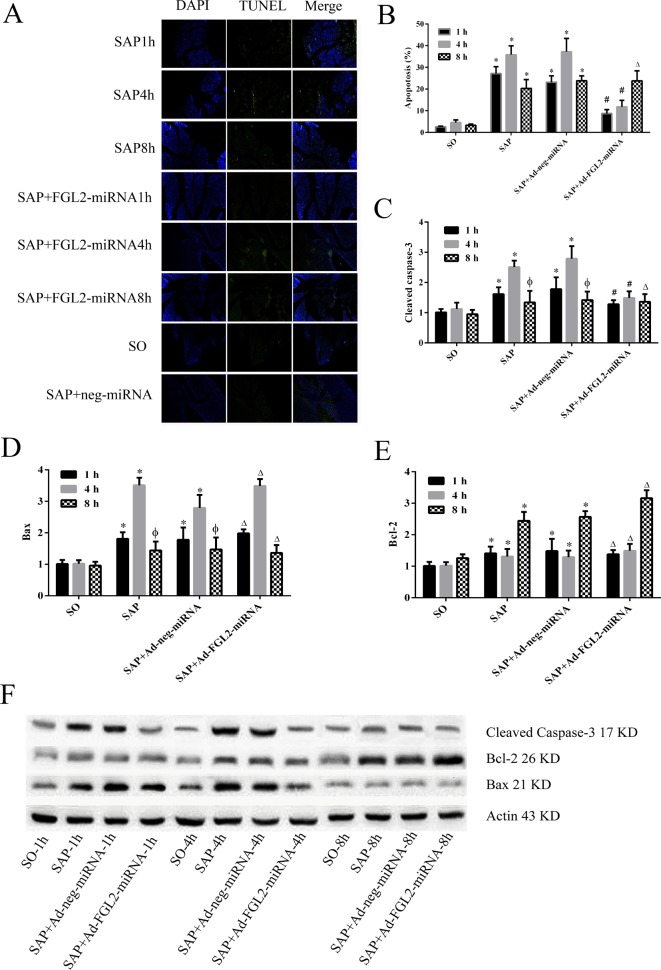
Effect of Ad-FGL2-miRNA on apoptosis of acinar cells in SAP The apoptosis of acinar cells was analyzed by TUNEL staining. (**A**) Apoptosis of acinar cells detected by TUNEL staining. (**B**) The frequencies of TUNEL-positive cell in pancreas. (**C**) Relative expression of cleaved caspase-3 protein in pancreas. (**D**) Relative expression of Bax protein in pancreas. (**E**) Relative expression of Bcl-2 protein in pancreas. (**F**) Relative expression of cleaved caspase-3, Bax, Bcl-2 protein expression detected by Western blotting. There was no difference amongst the three time points in the SO group (*P*>0.05). The data are expressed as the mean ± S.D. *P<0.01 compared with SO group at respective time point; ^#^*P*<0.01 compared with SAP or SAP + Ad-negative-miRNA group at each time point; ^ϕ^*P*>0.05 compared with SO group at each time point; ^∆^*P*>0.05 compared with SAP or SAP + Ad-negative-miRNA group at each time point.

The apoptosis of acinar cells was analyzed by TUNEL staining. As shown in Figure 6, the number of TUNEL-positive cells was increased at 1 and 4 h post SAP induction, whereas the apoptotic cells were decreased in SAP mice at 8 h. Moreover, the TUNEL-positive cells were dramatically reduced in Ad-FGL2-miRNA-treated mice at 1 and 4 h post SAP induction. Nonetheless, Ad-FGL2-miRNA appeared to have no significant impact on SAP mice at 8 h. There was no significant change in both SAP and SAP + Ad-neg-miRNA groups. Also, further experiments showed that there was significant decreased cleavage of caspase-3 in Ad-FGL2-miRNA-treated mice at 1 and 4 h post SAP induction. In addition, Ad-FGL2-miRNA did not affect cleavage of caspase-3 at 8 h post SAP induction. However, Ad-FGL2-miRNA did not have influence on the expression of certain apoptosis-related proteins, such as proapoptotic protein (Bax) and anti-apoptotic protein (Bcl-2) (Figure 6).

### TLR4 and p38 MAPK were involved in the process of *FGL2* gene silencing

**Figure 7:**
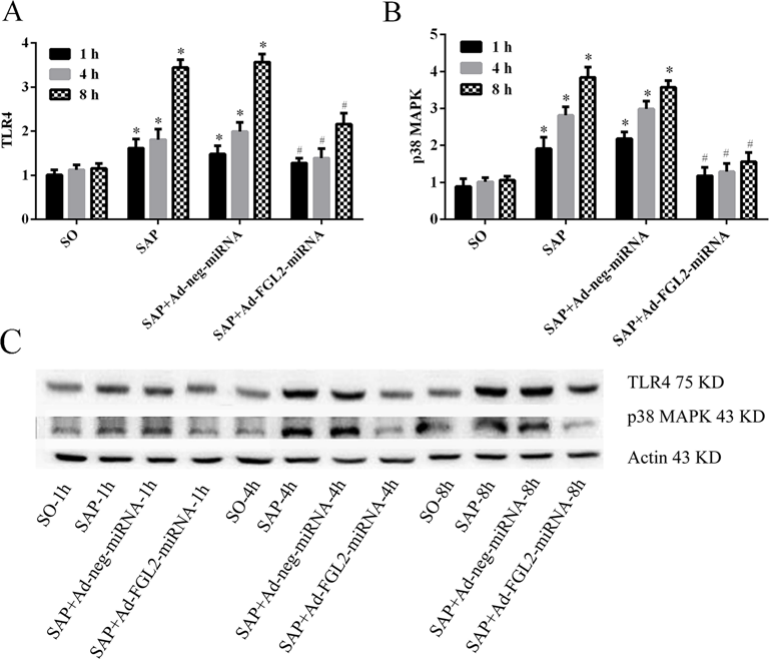
Effect of Ad-FGL2-miRNA on the expressions of TLR4 and p38 MAPK protein in SAP (**A**) Relative expression of TLR4 protein in pancreas; (**B**) Relative expression of p38 MAPK protein in pancreas; (**C**) Relative expression of TLR4 and p38 MAPK protein expression detected by Western blotting. There was no difference amongst the three time points in the SO group (*P*>0.05). The data are expressed as the mean ± S.D. *P<0.01 compared with SO group at respective time points; ^#^*P*<0.01 compared with SAP or SAP + Ad-negative-miRNA group at each time point.

The TLR4 and p38 MAPK protein have been shown to play a pivotal role in the development of SAP, inducing the release of several inflammatory cytokines [4]. To investigate the role that FGL2 up-regulation plays in TLR4 and p38 MAPK activation, Ad-FGL2-miRNA was applied to deplete FGL2 expression in SAP mice. As shown in Figures 3 and 7, the gradual up-regulation of FGL2 level was consistent with the TLR4 and p38 MAPK signaling activation in SAP mice. After *FGL2* gene silencing with Ad-FGL2-miRNA, the levels of TLR4 and p38 MAPK were significantly decreased (*P*<0.01; Figure 7). Our results apparently revealed the potential mechanisms that FGL2 plays as a co-activator in inducing the TLR4 and p38 MAPK signaling pathways in SAP mice.

## Discussion

SAP is characterized as an autodigestive and progressive inflammatory disorder with a high mortality rate that ranges from 10 to 30% [4]. No intervention strategies exist, and effective therapies have not been developed due to the elusive understanding of SAP pathogenesis. Therefore, it is of importance to investigate the pathophysiology and potential therapeutic strategies for SAP.

Apoptosis and necrosis are closely related with the pathogenesis of SAP. In the early stage of SAP, various factors could induce pancreatic acinar cell injury/death; therefore acinar cell necrosis would increase pancreatic intracellular activity of trypsin, releasing various inflammatory cytokines and activating inflammation cascade. However, apoptosis of acinar cells would reduce the release and activity of trypsin and ameliorate inflammation cascade. Hence, it is generally accepted that shifting death responses from necrosis to apoptosis has a therapeutic implication [1,20,21].

Although several lines of evidence have shown that FGL2 is closely linked to apoptosis in several other diseases [15,22,23], its role in the regulation of cell death and inflammatory response during SAP remains unclear. In this study, we for the first time used *in vivo* gene knockdown model to investigate the role of FGL2 in SAP mice. We have confirmed that *FGL2* gene silencing could remarkably alleviate the severity of SAP and inhibit against acinar cell apoptosis at the early stage of the disease by modulating cleaved caspase-3 expression (Figure 6). FGL2 expression was inhibited using an *in vivo* adenovirus-mediated transfer, and its effects on the release of inflammatory cytokines in sodium taurocholate-induced SAP mice were assessed. Neutrophil infiltration in the pancreas was recognized as parameter of pancreatic injury [24]. Our observation demonstrated that *FGL2* gene silencing with Ad-FGL2-miRNA substantially alleviated the pancreatic damage, as shown by the results of histological examination, leukocyte infiltration, and serum amylase (Figures 1 and 2). In addition, it was observed that Ad-FGL2-miRNA significantly down-regulated the expression levels of proinflammatory cytokines (TNF-α and IL-1β) in the pancreas (Figure 5), which played a pivotal role in local tissue injury developing to the systemic inflammatory responses in SAP [20]. The decreased levels of proinflammatory cytokines were consistent with the severity of pancreatic injury as indicated by histological characteristic. Moreover, we measured several apoptosis-related proteins by Western blot. We found that Ad-FGL2-miRNA dramatically reduced the expression of cleavage of caspase-3, whereas the levels of Bax and Bcl-2 were not affected (Figure 6). Therefore, the present study strongly suggests that FGL2-targetted miRNA treatment of SAP mice holds promising therapeutic potential.

It is worth noting that the effect of *FGL2* gene silencing (Ad-FGL2-miRNA) was significantly remarkable at 1 and 4 h post SAP induction; however, we did not observe such phenomenon at 8 h. We consider that there might be several reasons for this phenomenon: first, an emerging concern is that RNAi monotherapy may not ultimately confine disease progress; also, as the concept that the therapeutic utility of shifting the cell death modality from necrosis to apoptosis is widely accepted, we infer that apoptosis was the major pathophysiological change at the early stage of disease development [3], while necrosis dominated disease progression in the later phase. Consequently, Ad-FGL2-miRNA probably did not have profound impact during the later stage of SAP.

We adopted RNAi technology to silence FGL2 expression. The successful application of chemically synthesized siRNAs, plasmid expression of shRNAs, or plasmid expression of miRNAs to silence target genes makes RNAi a powerful means for studying gene function and may be a rational therapeutic approach for a variety of diseases [15,25–27]. It is essential for gene therapy that a suitable vector is used *in vivo*. Based on this consideration, plasmid-based shRNAs are unlikely to be appropriate for use in primates due to the short half-life, low *in vivo* transfection efficiency, and necessary assistance by hydrodynamic injection. To conquer these defects, adenoviral vectors are developed. Replication deficient-recombinant adenovirus is one of the most advanced and best-studied vector systems that is capable of expressing exogenous genes at a high level [28]. They are highly efficient at transferring genes to various target cells, including both dividing and nondividing cells. Expectedly, Ad-FGL2-miRNA exhibited remarkable inhibitory effects on FGL2 expression at both mRNA and protein levels.

In the present study, we measured the expression of several apoptosis-related proteins in pancreas (cleaved caspase-3, Bax, and Bcl-2). Caspase-3 is known to play a key role in the execution of apoptosis. Caspase-3 activation by cell death signals may happen via three pathways: the mitochondrial (related to Bax and Bcl-2), the endoplasmic reticulum, or a death receptor (FAS, FAS-L) [29–32]. Bcl-2 is a gene located at chromosome 18q21 that encodes a 26-kDa protein and blocks programmed cell death without affecting cellular proliferation. The Bax protein that belongs to the Bcl-2 family promotes apoptosis. The initiation of apoptosis is governed by the equilibrium between Bax and Bcl-2 [33]. Our findings have shown that there was significantly decreased cleavage of caspase-3 in Ad-FGL2-miRNA group, while Bax and Bcl-2 were not affected. This is consistent with a prior study investigating fulminant hepatic failure [15], whereas it is different from one study investigating diabetes in rats [22]. Therefore, it remains to be concluded whether FGL2 presents different biological mechanisms in various diseases.

Increasing studies in the recent years have provided strong evidence in supporting an essential role of TLR4 and p38 MAPK signaling in the pathogenesis of SAP [4]. In the present study, we demonstrated that *FGL2* gene silencing with Ad-FGL2-miRNA could significantly and simultaneously down-regulate the expression level of TLR4 and p38 MAPK, providing strong evidence that FGL2 is involved in these signaling pathways.

In summary, this is the first report that gene silencing of FGL2 inhibits apoptosis and protects against sodium taurocholate-induced SAP in mice by modulating cleaved caspase-3, TLR4, and p38 MAPK. Our results with this efficient knockdown system may extend our knowledge of gene therapy. Although the preliminary data are very encouraging, it is still needed to be well discussed and further study should definitely be continued.

## Abbreviations

Ad-FGL2-miRNA: adenovirus-mediated artificial miRNA targetting FGL2
FGL2: fibrinogen-like protein 2
HRP: horseradish peroxidase
IL-1β: interleukin-1β
MAPK: mitogen-activated protein kinase
SAP: severe acute pancreatitis
TNF: tumor necrosis factor-α
TLR4: toll-like receptor 4
Treg: regulatory T cell
TUNEL: terminal deoxynucleotidyl transferase mediated dUTP-biotin nick-end labeling
